# Admission prognostic nutritional index improves risk stratification for the carbapenem-resistant phenotype in culture-positive *Klebsiella pneumoniae*: a retrospective cohort study

**DOI:** 10.3389/fcimb.2026.1828441

**Published:** 2026-08-06

**Authors:** Kaixuan Zhang, Huihui Li, Yuanyuan Xu, Sudi Zhu, Mengyu Zhang, Henggui Hu, Shuguo Qin, Pingping Zhao

**Affiliations:** Department of Clinical Laboratory, Wanbei Coal Electric Group General Hospital, Suzhou, China

**Keywords:** carbapenem-resistant phenotype, prognostic nutritionalindex, risk stratification, implementation, infection prevention and control

## Abstract

**Background:**

Carbapenem-resistant *Klebsiella pneumoniae* (CRKP) remains an infection-control challenge. We assessed whether admission immuno-nutritional status, measured by the Prognostic Nutritional Index (PNI), is associated with the CRKP phenotype and adds risk information beyond clinical exposures.

**Methods:**

We conducted a retrospective cohort study at Wanbei Coal Electricity Group General Hospital, China (2020–2025). Among 2,571 inpatients with a positive *K. pneumoniae* culture, 1,360 adults with a first positive culture obtained >48 hours after admission were included and chronologically divided into a derivation cohort (n=987) and a temporal internal validation cohort (n=373). PNI was calculated from laboratory tests obtained within 24 hours of admission. The intended prediction window was after *K. pneumoniae* identification from the index culture but before antimicrobial susceptibility results were finalized; all predictors were restricted to information available by the index specimen-collection date. CRKP was defined as non-susceptibility to at least one carbapenem. Multivariable logistic regression was used for model development, and performance was assessed by area under the curve (AUC), calibration, and reclassification indices.

**Results:**

In the derivation cohort, 408 patients (41.3%) had the CRKP phenotype. Lower admission PNI was independently associated with CRKP (adjusted odds ratio [OR] 0.97 per 1-point increase; 95% confidence interval [CI] 0.96–0.98; *P* < 0.001). Adding PNI to the clinical model increased AUC from 0.690 to 0.708 in the derivation cohort and from 0.682 to 0.700 in the temporal internal validation cohort. Reclassification also improved (NRI 0.230 [95% CI 0.130–0.352]; IDI 0.014 [95% CI 0.005–0.028]). Observed CRKP proportions were 20.4%, 45.9%, and 65.6% across descriptive low-, intermediate-, and high-risk tiers. In an extended sensitivity model adjusting for admission-to-culture interval and specimen source, PNI remained independently associated with CRKP.

**Conclusions:**

Admission PNI was independently associated with the CRKP phenotype and provided modest incremental risk information among adults with hospital-onset, culture-positive *K. pneumoniae*. The model is descriptive and hypothesis-generating and requires external validation before clinical use.

## 1 Introduction

Carbapenem-resistant *Klebsiella pneumoniae* (CRKP) remains a major challenge for hospital infection prevention and control (IPC) (Verdugo-Paiva et al., 2022). Once established, CRKP can spread through patient contacts and shared care environments, creating clusters that are difficult to contain. Effective containment depends on timely identification and appropriate precautions, yet universal screening is rarely feasible in routine practice (Gomides et al., 2022). A recurring operational question for IPC teams is how to stratify the likelihood of resistant phenotypes once hospital-onset, culture-positive *Klebsiella pneumoniae* (*K. pneumoniae*) has been identified but before antimicrobial susceptibility results are finalized (Wangchinda et al., 2022; Orena et al., 2024).

Most risk assessments rely on “extrinsic” exposures—such as recent antibiotic use, intensive care unit (ICU) admission, and invasive devices. While these factors reflect selection pressure and care intensity, they do not fully explain observed heterogeneity: among patients with broadly similar exposure histories, only a subset is found to yield a carbapenem-resistant phenotype (Aslan et al., 2024). This pattern suggests that intrinsic host resilience may provide additional descriptive information beyond exposure checklists alone (Wielders et al., 2022).

Immuno-nutritional status offers a practical window into host resilience. Nutritional reserves and immune competence influence barrier function and microbial clearance, which may affect persistence of resistant organisms under antibiotic pressure (Barazzoni et al., 2022; Ji et al., 2025). The Prognostic Nutritional Index (PNI), derived from serum albumin and lymphocyte count, is available from routine admission laboratory tests and can be calculated without additional cost (Capurso et al., 2024). Although PNI is widely used for prognostic stratification in oncology and surgery, its value for risk stratification of carbapenem-resistant phenotypes in hospital-onset, culture-positive *K. pneumoniae* has not been well defined (Nergiz and Ozturk, 2023).

Distinct from studies that predict infection onset among all inpatients, this investigation focuses on resistance phenotype. We studied adults with hospital-onset, culture-positive *K. pneumoniae* and assessed whether immuno-nutritional depletion at admission is associated with a higher likelihood of the CRKP phenotype compared with the carbapenem-susceptible phenotype (CSKP). We hypothesized that lower admission PNI reflects a vulnerable host state linked to the resistant phenotype, independent of conventional exposure factors (Cai et al., 2024; Efthimiou et al., 2024).

Accordingly, we conducted a hospital-wide retrospective cohort study with three aims: (i) to quantify the independent association between admission PNI and the CRKP phenotype; (ii) to assess whether adding PNI improves risk stratification beyond exposure-based models; and (iii) to explore potential heterogeneity of this association between medical and surgical inpatients. By integrating admission host data with established exposures, we aimed to develop a descriptive phenotype-classification model for adults with hospital-onset, culture-positive *K. pneumoniae*, rather than a general inpatient screening tool for future CRKP acquisition.

## 2 Materials and methods

### 2.1 Study design and ethical considerations

We conducted a retrospective cohort study at Wanbei Coal Electricity Group General Hospital, China. The study period spanned from 2020 to 2025. The research protocol adhered to the principles of the Declaration of Helsinki and was approved by the Ethics Committee of Wanbei Coal Electricity Group General Hospital, China (Approval No.: 2025-131). Given the retrospective design and the use of anonymized data, the requirement for informed consent was waived by the institutional review board.

### 2.2 Participant screening and eligibility

**Figure 1:**
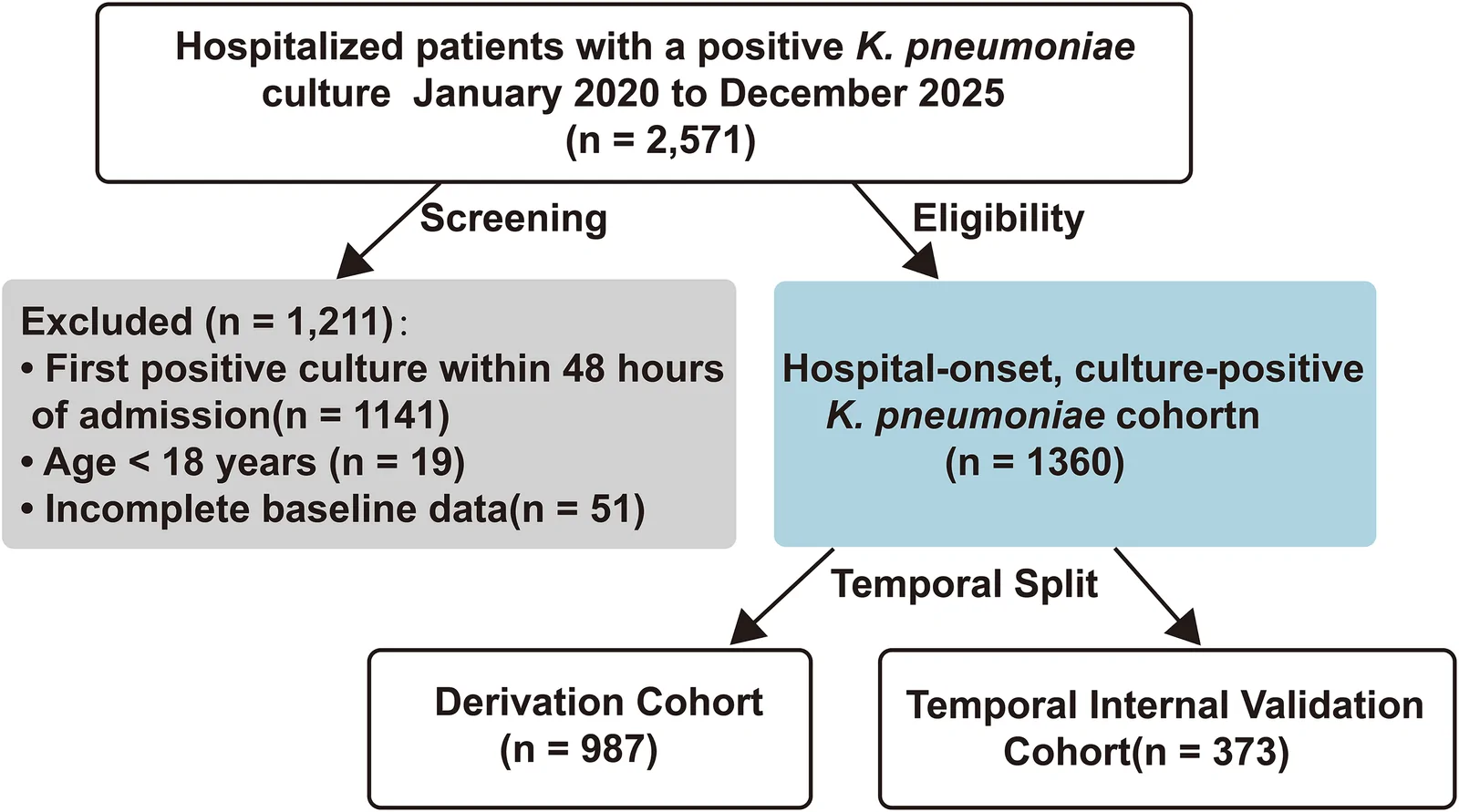
Flowchart of patient selection and study design. The initial screening identified 2,571 unique hospitalized patients with a positive *K. pneumoniae* culture. Duplicate isolates were removed by retaining only the first isolate per patient during the study period. In total, 1,211 patients were excluded: 19 pediatric patients (<18 years), 1,141 patients whose first positive culture was obtained ≤48 hours after admission, and 51 patients with incomplete baseline data required to calculate PNI. The final cohort comprised 1,360 patients with hospital-onset, culture-positive *K. pneumoniae* and was chronologically divided into a derivation cohort (n=987) and a temporal internal validation cohort (n=373).

Data were retrieved from the hospital’s Electronic Medical Record (EMR) system. During data extraction, duplicate isolates were removed and only the first positive *K. pneumoniae* isolate per patient during the study period was retained; therefore, all screened records represented unique patients. We initially identified 2,571 hospitalized patients with a positive *K. pneumoniae* culture (culture positivity may represent colonization or infection depending on specimen type and clinical context). To define an adult hospital-onset, culture-positive cohort (**Figure 1**), we applied a three-step exclusion process. First, 19 pediatric patients (<18 years) were excluded. Second, patients whose first positive culture was obtained ≤48 hours after admission (n=1,141) were excluded as not meeting the hospital-onset definition. Hospital-onset culture positivity was defined as a first positive culture obtained >48 hours after admission; this timing-based definition does not establish that the organism was acquired during hospitalization, because pre-admission colonization detected later cannot be excluded. Finally, 51 patients were excluded due to missing baseline laboratory data required for PNI calculation (specifically albumin or lymphocyte counts measured on admission). After screening, 1,360 eligible patients were included. The cohort was chronologically split into a derivation cohort (the first 987 consecutive eligible admissions) and a temporal internal validation cohort (the subsequent 373 admissions) according to admission date. Because the primary outcome was the carbapenem-resistant phenotype among culture-positive patients rather than infection-related clinical outcomes, we did not perform formal adjudication of colonization versus infection.

### 2.3 Microbiological identification and susceptibility testing

Species identification was confirmed by MALDI-TOF MS (Bruker microflex LT/SH). Antimicrobial susceptibility testing was primarily performed using the VITEK 2XL Compact system. When a carbapenem susceptibility result was unavailable from the VITEK 2XL system, the Kirby–Bauer disk diffusion method was used for that result. Susceptibility interpretations were based on the CLSI criteria documented by the laboratory at the time of testing. CRKP was defined as non-susceptibility (intermediate or resistant) to at least one carbapenem agent (imipenem, meropenem, or ertapenem). *E. coli* ATCC 25922 and *K. pneumoniae* ATCC 700603 were used as quality control strains. No molecular testing was performed to detect carbapenemase genes or other resistance mechanisms.

### 2.4 Data extraction and variable definitions

Clinical data were extracted from the EMR system. Baseline demographic and immuno-nutritional variables were obtained from the first laboratory tests within 24 hours of hospital admission. The index date was the specimen-collection date of the first hospital-onset K. pneumoniae-positive culture. The intended prediction window was after K. pneumoniae identification from the index culture but before antimicrobial susceptibility results were finalized. All predictor information was restricted to data available before or on the index date. The six predictors in the primary model were age, admission PNI, admission service, recent antibiotic exposure, ICU admission, and mechanical ventilation; specimen source and admission-to-culture interval were additionally available for the extended sensitivity model. The model was therefore not an admission-only model and was not designed to predict future acquisition of CRKP. Laboratory parameters included white blood cell count (WBC), platelet count (PLT), C-reactive protein (CRP), serum albumin, and the neutrophil-to-lymphocyte ratio (NLR). PNI was calculated as: PNI = Albumin (g/L) + 5 × Total Lymphocyte Count (10^9/L). The C-reactive protein/Albumin Ratio (CAR) was calculated as serum CRP (mg/L) divided by albumin (g/L). Recent antibiotic exposure was defined as receipt of systemic antibiotics within 30 days before the index date. ICU admission and mechanical ventilation were recorded only if they occurred before or on the index date. Information after the index date was not used. The admission-to-culture interval was calculated as the number of days between hospital admission and collection of the first K. pneumoniae-positive specimen. Specimen sources were classified as sputum, blood, urine, bronchoalveolar lavage fluid, or other specimens. The EMR/LIS did not contain a consistently recorded field distinguishing cultures obtained for clinical diagnosis from those obtained for active surveillance. The broad antibiotic-exposure variable did not distinguish antibiotic class, dose, or duration.

### 2.5 Statistical analysis

Continuous variables were compared using Student’s t test or the Mann–Whitney U test, as appropriate. Categorical variables were compared using the chi-square test or Fisher’s exact test. We assessed the functional form of PNI using restricted cubic splines (RCS). Because the non-linearity test was not significant, PNI was modeled as a continuous variable. For model development, candidate predictors were screened using least absolute shrinkage and selection operator regression (LASSO) with 10-fold cross-validation. Variables with non-zero coefficients were entered into multivariable logistic regression to estimate associations with the CRKP versus CSKP phenotype. In line with the Transparent Reporting of a multivariable prediction model for Individual Prognosis Or Diagnosis statement (TRIPOD) (Collins et al., 2015), the full model specification (intercept and coefficients) is provided in **Supplementary Table 1**. A total of 18 candidate predictors were prespecified based on clinical plausibility, prior literature, and data availability in the EMR. In the derivation cohort, 408 patients had the CRKP phenotype, and the final multivariable model retained 6 predictors, corresponding to an approximate events-per-variable ratio of 68. A nomogram was constructed from the final model. Discrimination was evaluated using ROC analysis and AUC. Calibration was assessed with calibration plots using 1,000 bootstrap resamples. We quantified calibration using the Brier score, calibration intercept, and calibration slope (**Supplementary Table 3**). Clinical utility was evaluated using decision curve analysis (DCA). Incremental value over the clinical exposure model was assessed using NRI and IDI. Predicted probabilities defined three descriptive risk tiers: low (<33%), intermediate (33–66%), and high (>66%). These cutoffs were used only to summarize observed risk gradients and were not validated intervention thresholds. To evaluate potential confounding by culture timing and specimen source, we fitted an extended sensitivity model that added the admission-to-culture interval, modeled per 7-day increase, and specimen source to the six-variable primary model; sputum was the reference specimen category. We assessed interaction by admission service using a multiplicative term (PNI × surgical admission). Sensitivity analyses included excluding patients with CRKP identified within 3 days of admission. As a robustness analysis, we performed propensity score matching (PSM); matching details and balance diagnostics are provided in **Supplementary Table 4**. We used complete-case analysis because few patients lacked baseline data needed for PNI calculation. E-values were calculated for the adjusted association in the PNI strata analysis (low PNI ≤38 vs high PNI ≥45; **Supplementary Table 5**) using the approach described by VanderWeele and Ding (EValue package in R). All analyses were conducted using R (version 4.2.1) and GraphPad Prism 9.0. A two-sided *P* value <0.05 was considered statistically significant.

## 3 Results

### 3.1 Baseline characteristics and cohort comparison

A total of 1,360 unique adult patients with hospital-onset, culture-positive *K. pneumoniae* met the inclusion criteria, comprising 987 (72.6%) in the derivation cohort and 373 (27.4%) in the temporal internal validation cohort. As shown in **Table 1**, the temporal internal validation cohort had a higher median age (71.0 vs. 67.0 years, *P* = 0.001), higher rates of mechanical ventilation (52.3% vs. 30.8%, *P* < 0.001) and surgical admission (42.6% vs. 26.2%, *P* < 0.001), and higher all-cause mortality (8.8% vs. 5.1%, *P* = 0.013). PNI (*P* = 0.429), albumin (*P* = 0.727), and NLR (*P* = 0.958) were comparable between cohorts. The admission-to-culture interval was also similar (9.61 [4.54–20.45] vs. 8.36 [4.01–20.96] days; *P* = 0.413), as was the specimen-source distribution (*P* = 0.165). These differences likely reflect temporal drift in case mix and practice patterns over the study period, although changes in culturing practices or other selection mechanisms cannot be fully excluded.

**Table 1:** Baseline characteristics of patients in the derivation and temporal internal validation cohorts.

Variables	Derivation cohort (n=987)	Temporal internal validation cohort (n=373)	*P* value
Demographics			
Age, years	67.0 (54.0-77.0)	71.0 (59.0-80.0)	0.001
Male sex	703 (71.2%)	267 (71.6%)	0.95
Comorbidities			
Hypertension	404 (40.9%)	216 (57.9%)	<0.001
Diabetes mellitus	64 (6.5%)	31 (8.3%)	0.289
COPD	24 (2.4%)	8 (2.1%)	0.912
Malignancy	54 (5.5%)	24 (6.4%)	0.582
Stroke	167 (16.9%)	78 (20.9%)	0.103
Clinical features			
Surgical Admission	259 (26.2%)	159 (42.6%)	<0.001
Mechanical ventilation	304 (30.8%)	195 (52.3%)	<0.001
Antibiotic exposure	947 (95.9%)	361 (96.8%)	0.577
ICU Admission	430 (43.6%)	148 (39.7%)	0.218
Culture timing and specimen source			
Admission-to-culture interval, days	9.61 (4.54-20.45)	8.36 (4.01-20.96)	0.413
Specimen source			0.165
Sputum	679 (68.8%)	256 (68.6%)	
Blood	55 (5.6%)	16 (4.3%)	
Urine	104 (10.5%)	35 (9.4%)	
Bronchoalveolar lavage fluid	31 (3.1%)	22 (5.9%)	
Other	118 (12.0%)	44 (11.8%)	
Laboratory markers			
WBC, ×10^9^/L	9.5 (6.8-13.2)	9.4 (6.8-13.3)	0.921
Neutrophil-to-Lymphocyte Ratio (NLR)	6.9 (3.4-13.7)	6.8 (3.5-12.4)	0.958
Platelets (PLT), ×10^9^/L	209.0 (156.0-268.0)	210.5 (155.8-257.0)	0.518
Albumin, g/L	36.6 (32.0-41.3)	36.6 (32.7-40.6)	0.727
C-reactive Protein (CRP), mg/L	43.5 (7.8-114.6)	35.6 (7.3-99.1)	0.126
Prognostic Nutritional Index (PNI)	42.1 (36.3-48.2)	42.7 (37.5-47.8)	0.429
CRP-to-Albumin Ratio (CAR)	0.4 (0.0-2.5)	0.1 (0.0-1.6)	<0.001
Outcomes			
Length of Stay, days	32.0 (18.0-55.0)	29.0 (18.0-55.0)	0.672
Mortality	50 (5.1%)	33 (8.8%)	0.013

Data are presented as median (interquartile range, IQR) for continuous variables and n (%) for categorical variables. *P* values were calculated using the Mann–Whitney U test or chi-square test, as appropriate. The admission-to-culture interval is reported in days. Specimen source was classified as sputum, blood, urine, bronchoalveolar lavage fluid, or other specimens. The *P* value for specimen source represents the overall chi-square test across the five categories; category-specific *post hoc P* values were not calculated.

### 3.2 Univariate analysis of risk factors for CRKP

In the derivation cohort, 408 (41.3%) patients had the CRKP phenotype. Univariate analysis (**Table 2**) showed that the CRKP group was older (70.0 vs. 65.0 years, *P* < 0.001) and had higher rates of ICU admission (55.4% vs. 35.2%, *P* < 0.001) and antibiotic exposure (97.5% vs. 94.8%, *P* = 0.048). The CRKP group had lower PNI (39.7 vs. 44.0, *P* < 0.001) and albumin (34.8 vs. 37.8 g/L, *P* < 0.001), together with higher NLR (8.8 vs. 5.7, *P* < 0.001) and CAR (1.0 vs. 0.1, *P* < 0.001). Surgical admission was less frequent in the CRKP group (18.4% vs. 31.8%, *P* < 0.001). The admission-to-culture interval was longer in the CRKP group (12.26 [6.29–22.51] vs. 7.57 [3.61–18.46] days; *P* < 0.001), and specimen-source distributions differed between groups (*P* < 0.001). Before multivariable modeling, we evaluated multicollinearity among the inflammatory and nutritional markers. The variance inflation factor (VIF) for PNI was 2.65, below the conservative threshold of 5; corresponding VIFs were 1.13 for CRP and 1.31 for NLR.

**Table 2:** Univariate analysis of risk factors for the CRKP phenotype in the derivation cohort.

Variables	CSKP group (n=579)	CRKP group (n=408)	*P* value
Demographics			
Age, years	65.0 (53.5-75.0)	70.0 (57.0-80.0)	<0.001
Male sex	399 (68.9%)	304 (74.5%)	0.066
Comorbidities			
Hypertension	236 (40.8%)	168 (41.2%)	0.948
Diabetes mellitus	43 (7.4%)	21 (5.1%)	0.193
COPD	10 (1.7%)	14 (3.4%)	0.133
Malignancy	39 (6.7%)	15 (3.7%)	0.052
Stroke	92 (15.9%)	75 (18.4%)	0.346
Clinical features			
Surgical Admission	184 (31.8%)	75 (18.4%)	<0.001
Mechanical ventilation	170 (29.4%)	134 (32.8%)	0.273
Antibiotic exposure	549 (94.8%)	398 (97.5%)	0.048
ICU Admission	204 (35.2%)	226 (55.4%)	<0.001
Culture timing and specimen source			
Admission-to-culture interval, days	7.57 (3.61-18.46)	12.26 (6.29-22.51)	<0.001
Specimen source			<0.001
Sputum	370 (63.9%)	309 (75.7%)	
Blood	36 (6.2%)	19 (4.7%)	
Urine	67 (11.6%)	37 (9.1%)	
Bronchoalveolar lavage fluid	15 (2.6%)	16 (3.9%)	
Other	91 (15.7%)	27 (6.6%)	
Laboratory markers			
WBC, ×10^9^/L	9.3 (6.5-13.1)	9.8 (7.3-13.5)	0.063
NLR	5.7 (2.8-11.2)	8.8 (4.5-16.0)	<0.001
Platelets (PLT), ×10^9^/L	211.5 (159.0-271.8)	205.0 (146.2-260.0)	0.238
Albumin, g/L	37.8 (32.8-42.3)	34.8 (30.6-39.0)	<0.001
CRP, mg/L	28.8 (3.6-97.6)	61.1 (17.7-140.5)	<0.001
PNI	44.0 (37.6-49.9)	39.7 (34.4-45.4)	<0.001
CAR	0.1 (0.0-1.8)	1.0 (0.1-3.4)	<0.001
Outcomes			
Length of Stay, days	29.0 (16.0-51.0)	35.0 (21.0-62.0)	<0.001
Mortality	27 (4.7%)	23 (5.6%)	0.589

Data are presented as median (interquartile range, IQR) for continuous variables and n (%) for categorical variables. *P* values were calculated using the Mann–Whitney U test or chi-square test, as appropriate. The admission-to-culture interval is reported in days. Specimen source was classified as sputum, blood, urine, bronchoalveolar lavage fluid, or other specimens. The *P* value for specimen source represents the overall chi-square test across the five categories; category-specific *post hoc P* values were not calculated.

### 3.3 Predictive feature screening and multivariable model construction

**Figure 2:**
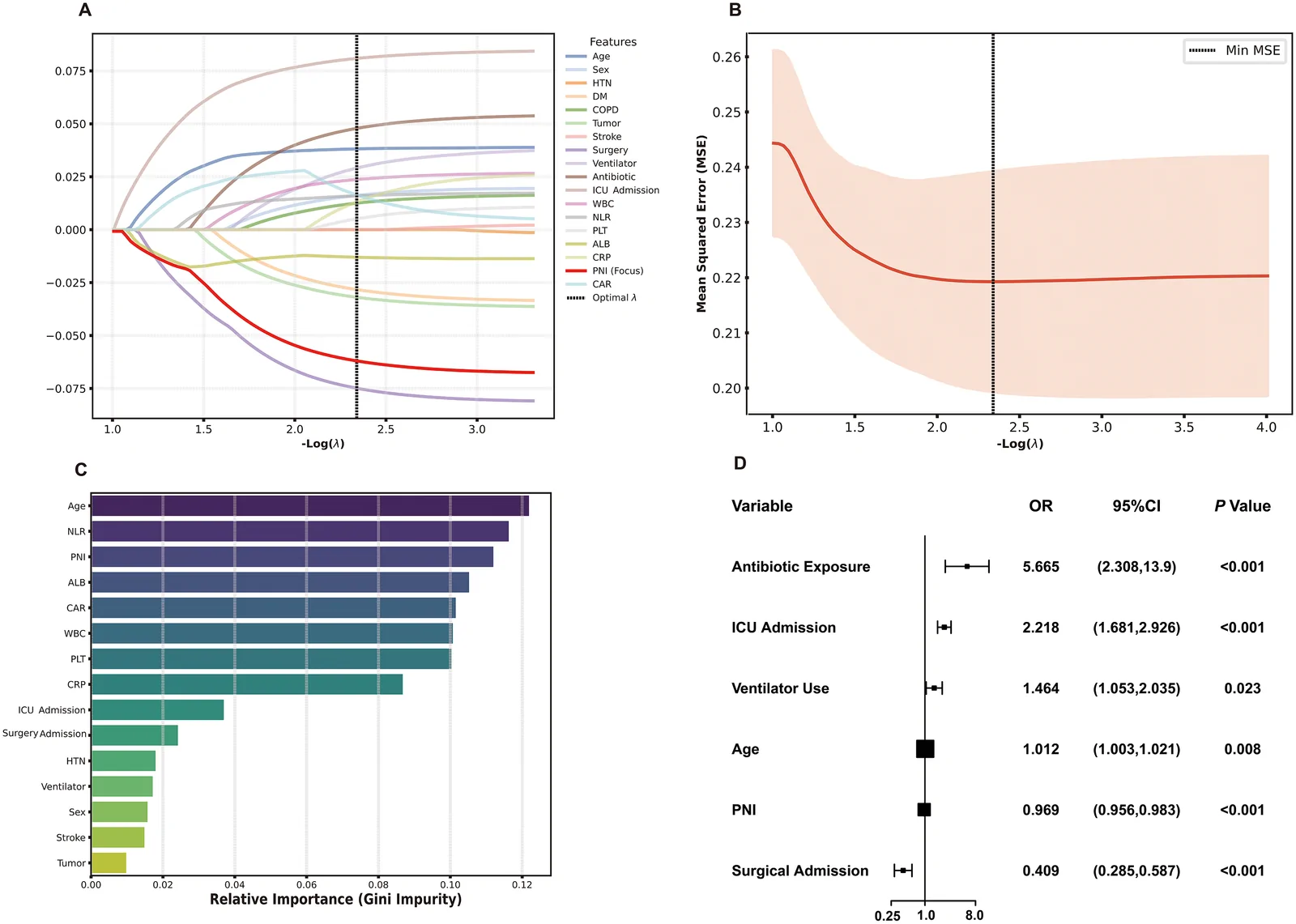
Feature screening and independent predictors. **(A)** LASSO coefficient paths across log(λ). **(B)** 10-fold cross-validation curve for λ selection; the dotted line indicates the optimal λ that minimizes mean squared error (MSE). **(C)** Random forest (RF) feature importance based on mean decrease in Gini. **(D)** Forest plot of independent predictors from multivariable logistic regression; squares indicate odds ratios (ORs) and lines indicate 95% confidence intervals (CIs). PNI is modeled as a continuous variable.

Feature screening combined penalized regression and ensemble learning. In the derivation cohort, LASSO selected 16 predictors at the optimal λ from 10-fold cross-validation (**Figures 2A, B**). PNI was retained among the selected predictors. In the random forest model, age and NLR ranked highest, and PNI ranked third by mean decrease in Gini (**Figure 2C**). Recent antibiotic exposure showed the largest effect size in multivariable logistic regression (OR 5.65; 95% CI 2.30–13.86; *P* < 0.001). ICU admission and mechanical ventilation remained associated with the CRKP phenotype (**Figure 2D**), and PNI was independently associated with the CRKP phenotype (OR 0.97 per 1-point increase; 95% CI 0.96–0.98; *P* < 0.001). In a categorical analysis of the total cohort using three PNI strata (≤38, 38–<45, and ≥45), lower PNI remained associated with higher odds of the resistant phenotype after adjustment [adjusted OR 1.83 (95% CI 1.37–2.45) for 38–<45 and 2.14 (95% CI 1.60–2.87) for ≤38, versus ≥45; **Supplementary Table 5**]. The corresponding E-values were 3.70 for the point estimate and 2.58 for the lower 95% confidence limit. Final multivariable logistic regression identified antibiotic exposure (OR 5.65, 95% CI 2.30–13.86), ICU admission (OR 2.22, 95% CI 1.68–2.93), mechanical ventilation (OR 1.46, 95% CI 1.05–2.03), age (OR 1.01, 95% CI 1.00–1.02), PNI (OR 0.97, 95% CI 0.96–0.98), and surgical admission (OR 0.41, 95% CI 0.28–0.59) as independent factors associated with the CRKP phenotype. In the extended sensitivity model additionally including admission-to-culture interval and specimen source, PNI remained independently associated with the CRKP phenotype (OR 0.97 per 1-point increase, 95% CI 0.95–0.98; *P* < 0.001). The admission-to-culture interval was not independently associated with the CRKP phenotype when modeled per 7-day increase (OR 1.03, 95% CI 0.99–1.09; *P* = 0.177). Compared with sputum, blood, urine, and bronchoalveolar lavage specimens were not independently associated with the CRKP phenotype, whereas the heterogeneous other-specimen category showed lower odds (OR 0.43, 95% CI 0.26–0.71; *P* = 0.001). The inverse association for surgical admission was attenuated but remained statistically significant (OR 0.47, 95% CI 0.32–0.68; *P* < 0.001). Full results of the primary and extended models are provided in **Supplementary Tables 1** and **S8**, respectively.

### 3.4 In-depth analysis of PNI: dose-response relationship and effect modification by clinical subtype

**Figure 3:**
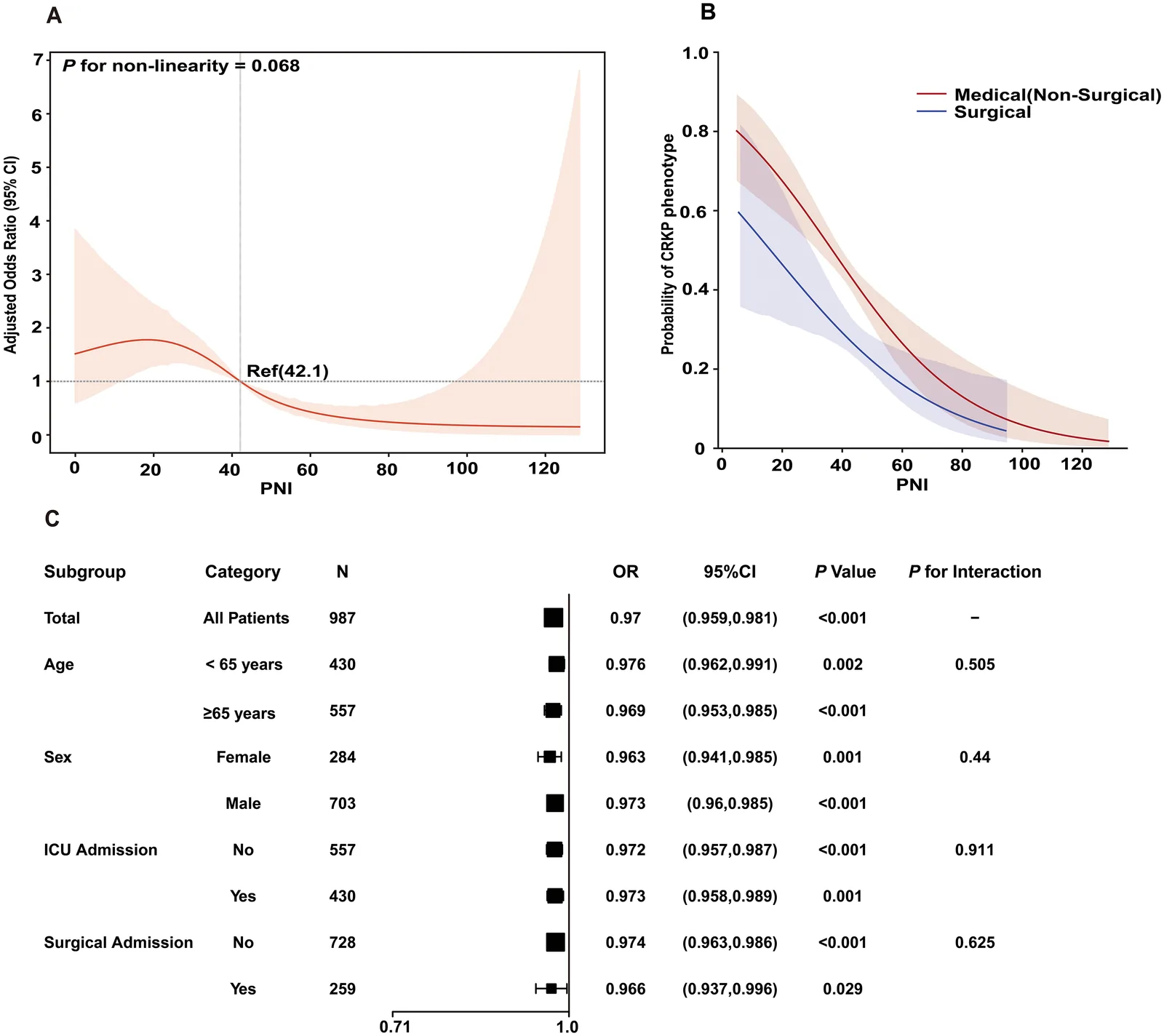
In-depth analysis of PNI: dose–response relationship and effect modification. **(A)** Dose–response relationship between PNI and CRKP phenotype modeled by restricted cubic splines (RCS); the line shows the adjusted OR and the shaded area indicates the 95% CI. The model was adjusted for age, recent antibiotic exposure, ICU admission, mechanical ventilation, and surgical admission. **(B)** Interaction plot of PNI versus predicted CRKP phenotype probability, stratified by admission service (medical vs surgical). Shaded areas indicate 95% CIs. **(C)** Subgroup analysis of the association between PNI and CRKP phenotype across prespecified strata; no significant interactions were observed (*P* for interaction >0.05).

Restricted cubic splines (RCS) were used to examine the dose–response pattern between PNI and the CRKP phenotype (**Figure 3A**). The test for non-linearity was not statistically significant (*P* for non-linearity = 0.068), indicating no strong evidence against an approximately linear association within the observed range. For parsimony and ease of interpretation, PNI was therefore modeled as a continuous linear predictor in the primary model. Categorized analyses showed a consistent gradient in the same direction. When stratified by admission service, PNI showed a similar inverse association in both medical and surgical cohorts (**Figure 3B**). The interaction test was not significant (*P* for interaction >0.05), indicating no evidence that the PNI effect differed by service. Subgroup analyses by age, sex, ICU admission, and recent surgery showed consistent effect estimates (**Figure 3C**), with odds ratios per 1-point higher PNI ranging from 0.96 to 0.98. No significant interactions were observed across strata (all *P* for interaction >0.05). Detailed model outputs are provided in **Supplementary Table 2**.

### 3.5 Sensitivity analysis: propensity score matching

**Figure 4:**
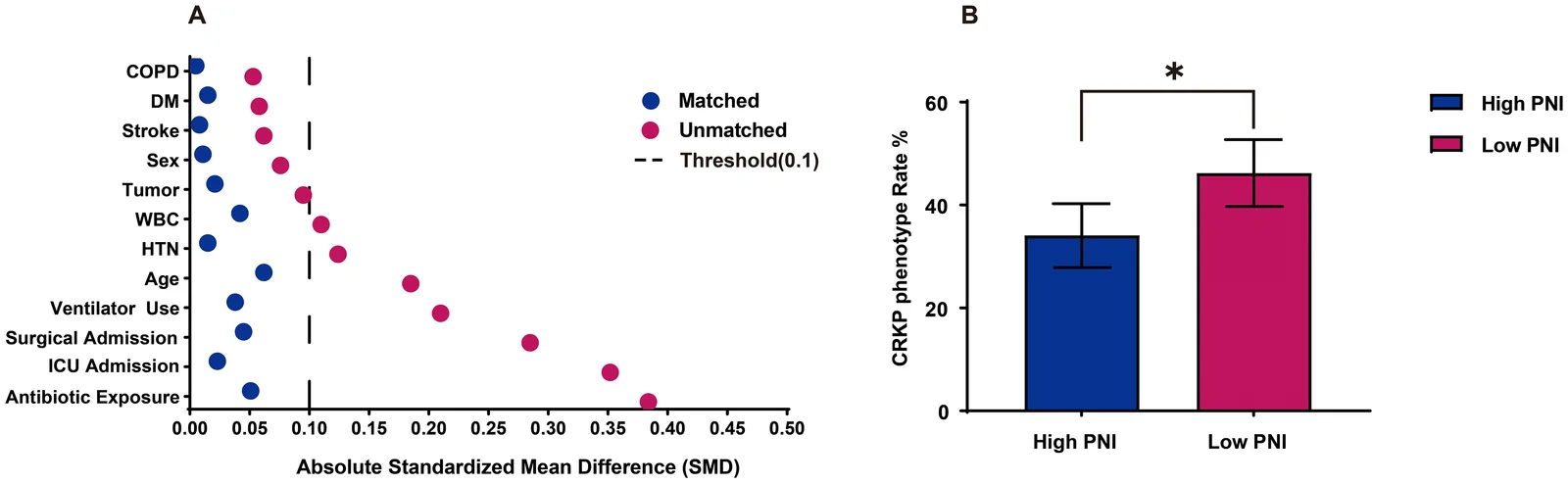
Sensitivity analysis using propensity score matching (PSM). **(A)** Covariate balance (Love plot) showing absolute standardized mean differences (SMDs) before and after matching for baseline covariates. **(B)** Observed CRKP phenotype proportion in the matched cohort (223 pairs), comparing low PNI (<42.1) versus high PNI (≥42.1). Because the CRKP phenotype is the outcome and was not used for matching, its post-match difference is expected. *P* = 0.012. The asterisk indicates P < 0.05.

Propensity score matching (PSM) was used to reduce baseline imbalance and to test robustness. Patients were grouped by the median PNI value (42.1) and matched 1:1, yielding 223 pairs (n=446). After matching, baseline covariates showed improved balance (**Figure 4A**). The CRKP phenotype was the outcome and was not included in the matching algorithm; its SMD (≈0.25) reflects the outcome difference between matched groups, not residual covariate imbalance. In the matched cohort, the observed CRKP phenotype proportion was higher in the low-PNI group than in the high-PNI group [46.2% (103/223) vs. 34.1% (76/223), *P* = 0.012; **Figure 4B**]. Residual confounding cannot be excluded.

### 3.6 Performance and clinical utility of the integrated predictive model

**Figure 5:**
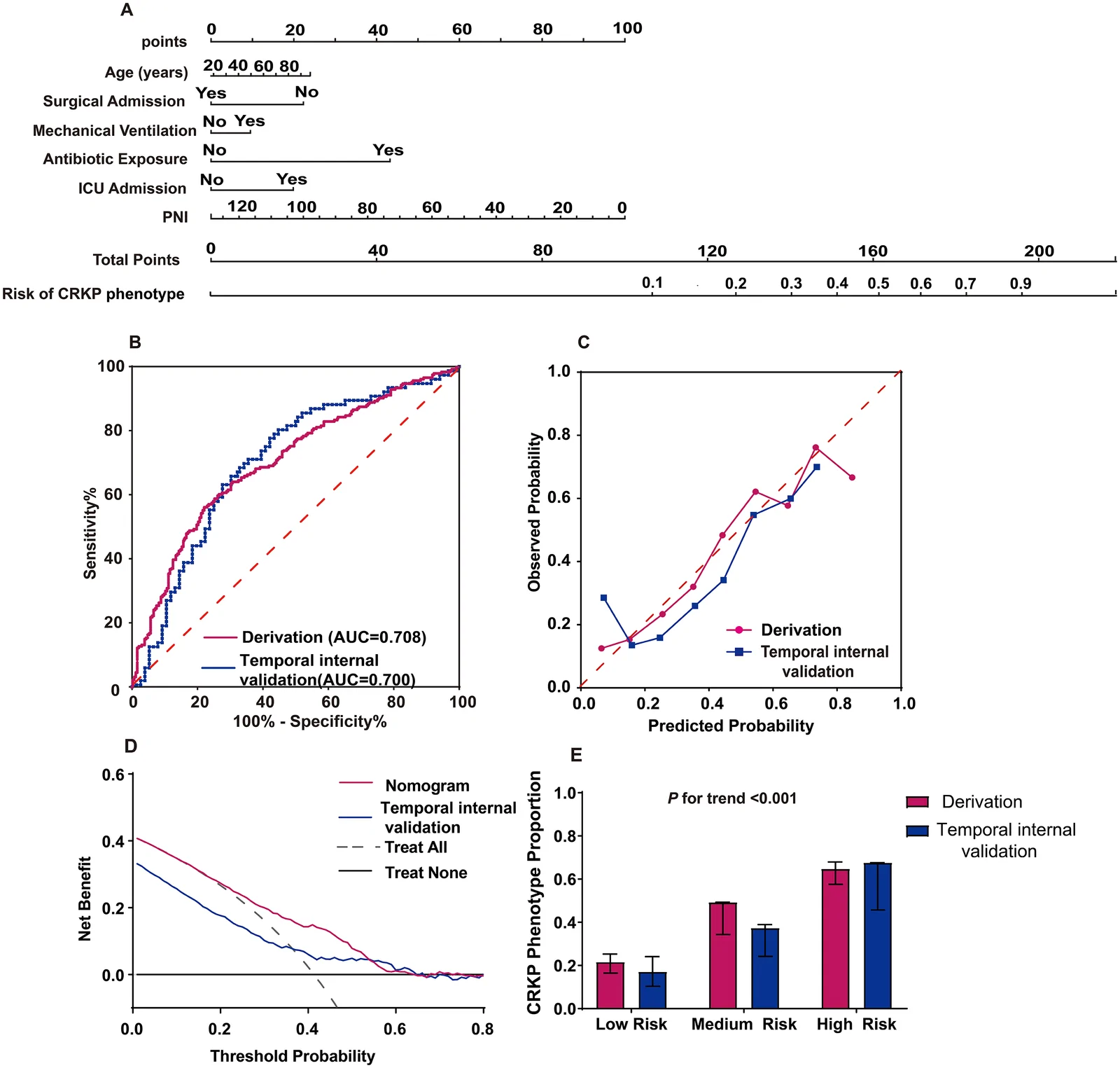
Nomogram and model evaluation. **(A)** Nomogram derived from the six-variable primary model. **(B)** ROC curves in the derivation and temporal internal validation cohorts (AUC 0.708 and 0.700). **(C)** Calibration plots in both cohorts. **(D)** Decision curve analysis in both cohorts. **(E)** Observed CRKP phenotype proportions across low (<33%), intermediate (33–66%), and high (>66%) descriptive predicted-risk tiers (*P* for trend <0.001). These tiers are descriptive and should not be interpreted as validated operational triage thresholds.

A nomogram was constructed from the final multivariable logistic regression model to estimate the probability of the CRKP phenotype (**Figure 5A**). The nomogram included age, PNI, antibiotic exposure, ICU admission, mechanical ventilation, and surgical admission. The Brier score was 0.213 in the derivation cohort and 0.204 in the temporal internal validation cohort (**Supplementary Table 3**). The temporal internal validation cohort had a calibration intercept of -0.333 and slope of 0.864. Discrimination was moderate and similar across cohorts (AUC 0.708 in the derivation cohort and 0.700 in the temporal internal validation cohort; **Figure 5B**). Calibration plots showed broadly aligned predicted and observed phenotype proportions (**Figure 5C**).

DCA suggested net benefit over treat-all and treat-none across a range of thresholds (**Figure 5D**). We defined three descriptive risk tiers from the nomogram-predicted probability: low (<33%), intermediate (33–66%), and high (>66%). In the temporal internal validation cohort, observed CRKP phenotype proportions increased from 17.1% in the low-risk group to 67.6% in the high-risk group (*P* for trend <0.001; **Figure 5E**). In the total cohort, the low-risk tier included 451 patients with 20.4% CRKP (92/451), the intermediate tier included 784 patients with 45.9% CRKP (360/784), and the high-risk tier included 125 patients with 65.6% CRKP (82/125). These strata summarize observed CRKP proportions within the study population and should not be interpreted as validated operational triage or stand-alone decision thresholds.

### 3.7 Incremental predictive value and risk stratification

Across modeling strategies, adding PNI to the clinical model yielded a modest but consistent improvement in discrimination. In the derivation cohort, AUC increased from 0.690 to 0.708 (ΔAUC 0.018), and in the temporal internal validation cohort from 0.682 to 0.700 (ΔAUC 0.018; **Table 3**). Reclassification metrics also favored the integrated model (NRI 0.230, IDI 0.014; both *P* < 0.001 in the derivation cohort; **Table 3**). When early-onset CRKP classifications were excluded, the integrated model showed similar AUC values (**Table 3**). These findings indicate modest incremental performance rather than a large increase in discrimination. PNI-stratified results are presented in **Supplementary Table 5**; admission-to-culture interval, specimen-source distributions, and the extended sensitivity model are presented in **Supplementary Table 7** and **S8**.

**Table 3:** Comprehensive evaluation of incremental predictive value and risk stratification.

Analysis scenarios	Derivation cohort (n=987)	Temporal internal validation cohort (n=373)	*P* value
A. Stepwise model comparison (AUC)			
Model 1: Clinical factors only^a^	0.690	0.682	–
Model 2: Integrated model (Clinical + PNI)	0.708	0.700	< 0.001^b^
B. Improvement metrics (Model 2 vs. Model 1)			
ΔAUC	0.018	0.018	–
Net reclassification improvement (NRI)	0.230 (0.130 – 0.352)	–	< 0.001
Integrated discrimination improvement (IDI)	0.014 (0.005 – 0.028)	–	< 0.001
C. Sensitivity analysis (excluding early-onset CRKP classifications)^c^			
Integrated Model AUC	0.699	0.697	–

Part A compares the discriminative ability (AUC) of the clinical baseline model and the PNI-integrated model in the derivation and temporal internal validation cohorts. Part B reports the incremental improvement of Model 2 versus Model 1 in the derivation cohort. Part C presents the sensitivity analysis excluding CRKP identified between 48 and 72 hours after admission. The PNI-only model is reported in **Supplementary Table 6**.

^a^The clinical model includes age, antibiotic exposure, ICU admission, mechanical ventilation, andsurgical admission. ^b^The P value was calculated using the DeLong test comparing Model 2 with Model 1. ^c^The sensitivity analysis excluded CRKP identified within 3 days after admission.

## 4 Discussion

Carbapenem-resistant *K. pneumoniae* remains a major threat to hospital infection prevention and control (IPC), largely because resistant phenotypes can persist and spread within clinical environments shaped by antibiotic pressure, invasive procedures, and host vulnerability (Wielders et al., 2022; Jung et al., 2023; Hassoun-Kheir et al., 2023). In this hospital-wide cohort of patients with hospital-onset, culture-positive *K. pneumoniae* (including colonization and infection), lower admission PNI was independently associated with the carbapenem-resistant phenotype after adjustment for established clinical exposures. The association remained stable after additional adjustment for admission-to-culture interval and specimen source. Adding PNI produced modest improvements in discrimination and reclassification, with overall moderate discriminative ability. The temporal internal validation cohort showed similar discrimination across admission periods, although this does not constitute external validation. Because predictors originated from different pre-susceptibility time points, the model should not be interpreted as an admission-only screening tool. Rather, it is a hypothesis-generating phenotype-classification model within the studied population.

Existing IPC frameworks primarily focus on extrinsic drivers of resistance, including prior antibiotic exposure, ICU stay, prolonged hospitalization, and invasive devices, which have been repeatedly associated with CRKP and other carbapenem-resistant Enterobacterales phenotypes (Aslan et al., 2024; Wielders et al., 2022; Jung et al., 2023; Hassoun-Kheir et al., 2023). Exposure history alone may not fully account for heterogeneity in detected resistance phenotypes. Our findings suggest that admission host condition—summarized by PNI—captures an additional dimension of vulnerability not reflected by exposure-based metrics alone (Li et al., 2025). Because PNI reflects physiological reserve at hospital entry rather than cumulative hospital exposures, it may provide supplementary descriptive information once hospital-onset, culture-positive *K. pneumoniae* has been identified (Chen et al., 2025; Du et al., 2023; Li et al., 2023).

The biological plausibility of this association is supported by evidence linking immuno-nutritional depletion to impaired host defense and altered microbial clearance. Hypoalbuminemia reflects systemic inflammation and physiological stress, while lymphopenia is a marker of impaired adaptive immune response and has been associated with adverse outcomes in severe infections and sepsis (Wang et al., 2022; Li et al., 2023). Malnutrition and immune dysfunction can affect mucosal barrier integrity and microbial persistence under antibiotic selection pressure. In this context, PNI offers a simple composite marker of host resilience that may influence whether resistant versus susceptible phenotypes are isolated (Ao et al., 2021). Subgroup analysis demonstrated that the predictive value of PNI remained consistent across both medical and surgical cohorts (*P* for interaction > 0.05). This suggests that the immuno-nutritional depletion captured by PNI reflects a fundamental host vulnerability that operates across admission services (Francis et al., 2023).

We also observed an inverse association between surgical admission and the CRKP phenotype. This pattern may reflect differences in case mix, sampling practices, and antibiotic exposure between services (Caudell et al., 2024; Wu et al., 2023). Surgical admissions may include more short-stay perioperative patients, whereas medical admissions may have a higher burden of prior colonization and repeated antimicrobial exposure. Culture indications and specimen types can also differ by service (Yang et al., 2023). In the extended sensitivity model, adjustment for specimen source attenuated but did not eliminate the inverse association for surgical admission, suggesting that culture source may partly, but not fully, account for this pattern. Because clinical and surveillance cultures could not be distinguished, residual selection bias remains possible (Salomão et al., 2017).

These findings should be interpreted cautiously in relation to infection prevention. Current IPC strategies emphasize early identification and targeted precautions for carbapenem-resistant organisms (Tomczyk et al., 2022; Guo et al., 2022). PNI is derived from routine admission laboratory tests and may provide supplementary phenotype-oriented information beyond exposure-based factors (Wang et al., 2019). However, the model applies only to patients with hospital-onset, culture-positive *K. pneumoniae* and is not intended as a universal admission screening tool. Given the moderate discriminative performance in temporal internal validation, the model should be viewed as a descriptive risk-enrichment approach rather than a stand-alone basis for IPC actions. The probability thresholds used in this study (<33%, 33–66%, and >66%) are descriptive categories rather than validated intervention cutoffs, and any clinical use would require independent validation and local calibration.

## 5 Limitations

This study has several limitations. First, this was a retrospective single-center study, and residual confounding cannot be excluded. Second, we did not formally adjudicate colonization versus infection. Because the outcome was the resistance phenotype among culture-positive patients rather than an infection-related clinical outcome, culture positivity may represent either colonization or infection. Third, the temporal internal validation cohort differed from the derivation cohort in case mix and showed mild miscalibration (calibration slope 0.864); this chronological split within one hospital does not constitute external validation. Fourth, no molecular testing was performed to identify carbapenemase genes or other resistance mechanisms. Fifth, retrospectively extracted EMR variables may have been incompletely captured or misclassified, and the antibiotic-exposure variable did not distinguish antibiotic class, dose, duration, cumulative exposure, or prior carbapenem use. Sixth, the model requires external multicenter and prospective validation. Seventh, predictors were not all anchored to admission: PNI was measured at admission, whereas several other predictors were defined up to the index culture date. Eighth, although specimen source was included in an extended sensitivity model, the EMR/LIS did not contain a consistently recorded field distinguishing clinical cultures from surveillance cultures. Ninth, prior CRKP/CRE carriage, recent hospitalization, interhospital transfer, ward location at culture, and illness-severity scores were not recorded in standardized extractable fields and therefore were not included in the analysis. Tenth, the LIS did not contain analyzable isolate-level fields identifying the susceptibility method used for each result, intermediate versus resistant categories, testing of all three carbapenems, or isolates classified solely through ertapenem non-susceptibility. Consequently, breakpoint harmonization across the entire study period and a resistant-only sensitivity analysis could not be evaluated. These limitations may have introduced residual confounding and microbiological misclassification.

## 6 Conclusions

In this single-center retrospective study, admission PNI was associated with the CRKP phenotype and provided modest incremental descriptive risk information among patients with hospital-onset, culture-positive *K. pneumoniae*. Its clinical utility remains to be established through external and prospective validation.

## Data Availability

The data analyzed in this study is subject to the following licenses/restrictions: The datasets used and/or analysed during the current study are not publicly available due to patient privacy and institutional restrictions. Requests to access these datasets should be directed to Kaixuan Zhang 2461530536@qq.com.
