# Supplementary material for: Admission prognostic nutritional index improves risk stratification for the carbapenem-resistant phenotype in culture-positive Klebsiella pneumoniae: a retrospective cohort study
Source: Front Cell Infect Microbiol. 2026 Aug 6;16:1828441. doi: 10.3389/fcimb.2026.1828441 (PMC13489799; doi:10.3389/fcimb.2026.1828441)
Supplement: Supplementary file 1 [file Table1.docx]

Supplementary Material

# Supplementary Data

Detailed quantitative parameters derived from the multivariable logistic regression model are provided below to support the findings presented in the main text. Table S1 reports the six-variable primary model in the derivation cohort.

Table S1. Detailed results of the primary multivariable logistic regression analysis.

| Variable | β Coefficient | Standard Error  (SE) | Wald χ2 | *P* Value | Odds Ratio  (95% CI) |
| --- | --- | --- | --- | --- | --- |
| Antibiotic exposure | 1.731 | 0.458 | 14.26 | <0.001 | 5.65 (2.30–13.86) |
| ICU Admission | 0.797 | 0.141 | 31.76 | <0.001 | 2.22 (1.68–2.93) |
| Ventilator Use | 0.379 | 0.169 | 5.02 | 0.025 | 1.46 (1.05–2.03) |
| Age | 0.012 | 0.005 | 6.96 | 0.008 | 1.01 (1.00–1.02) |
| PNI | -0.031 | 0.007 | 20.21 | <0.001 | 0.97 (0.96–0.98) |
| Surgical admission | -0.893 | 0.185 | 23.31 | <0.001 | 0.41 (0.28–0.59) |
| Constant | -1.781 | 0.509 | 12.24 | 0.001 | — |

Note: Variables were selected using penalized regression and ensemble learning, followed by multivariable logistic regression. The odds ratio (OR) for PNI reflects the change in odds per 1-unit increase. β, regression coefficient; SE, standard error; CI, confidence interval. Predicted probability for the CRKP phenotype was calculated as logit(P) = β0 + Σ(βi × Xi). ICU admission, ventilator use, and surgical admission were coded as 1 = yes and 0 = no. Age was modeled in years. PNI = albumin (g/L) + 5 × total lymphocyte count (10^9/L). Outcome: 1 = CRKP and 0 = CSKP.

Table S2. Subgroup analysis of the association between PNI and the CRKP phenotype.

| Subgroup | Category | N | OR | Lower CI | Upper CI | *P* Value | *P* for Interaction |
| --- | --- | --- | --- | --- | --- | --- | --- |
| Total | All Patients | 987 | 0.97 | 0.959 | 0.981 | <0.001 | - |
| Age | < 65 years | 430 | 0.976 | 0.962 | 0.991 | 0.002 | 0.505 |
|  | ≥ 65 years | 557 | 0.969 | 0.953 | 0.985 | <0.001 |  |
| Sex | Female | 284 | 0.963 | 0.941 | 0.985 | 0.001 | 0.44 |
|  | Male | 703 | 0.973 | 0.96 | 0.985 | <0.001 |  |
| ICU Admission | No | 557 | 0.972 | 0.957 | 0.987 | <0.001 | 0.911 |
|  | Yes | 430 | 0.973 | 0.958 | 0.989 | 0.001 |  |
| Surgical admission | No | 728 | 0.974 | 0.963 | 0.986 | <0.001 | 0.625 |
|  | Yes | 259 | 0.966 | 0.937 | 0.996 | 0.029 |  |

Note: Stratified analyses evaluated the consistency of the association between PNI and the CRKP phenotype across prespecified clinical subgroups. *P* for interaction was calculated using a likelihood ratio test comparing models with and without the interaction term (PNI × subgroup).

Calibration of the primary model was evaluated using quantitative metrics. As shown in Supplementary Table S3, the Brier score was 0.213 in the derivation cohort and 0.204 in the temporal internal validation cohort. The temporal internal validation cohort had a calibration intercept of -0.333 and a calibration slope of 0.864.

Table S3. Quantitative calibration metrics of the primary phenotype-classification model.

| Cohort | Number of Patients  (n) | Brier Score | Calibration Intercept | Calibration Slope |
| --- | --- | --- | --- | --- |
| Derivation Cohort | 987 | 0.213 | 0 | 1 |
| Temporal Internal Validation Cohort | 373 | 0.204 | -0.333 | 0.864 |

Note: In the derivation cohort, the intercept of 0 and slope of 1 represent the apparent reference calibration of the fitted model in the development dataset. The temporal internal validation cohort provides the chronological internal assessment; it is not an external validation cohort.

We performed propensity score matching (PSM) to reduce baseline imbalance. Patients in the derivation cohort were dichotomized into low-PNI (<42.1) and high-PNI (≥42.1) groups using the median PNI value. Propensity scores were estimated using age, sex, surgical admission, mechanical ventilation, recent antibiotic exposure, and ICU admission. We applied 1:1 nearest-neighbor matching without replacement with a caliper of 0.1 standard deviations of the logit of the propensity score. After matching, 223 pairs (n=446) were identified. The CRKP phenotype was the outcome and was not included in the matching algorithm. In the matched cohort, the CRKP phenotype proportion was higher in the low-PNI group than in the high-PNI group (46.2% vs. 34.1%; *P* = 0.012).

Table S4. Baseline demographic and clinical characteristics after 1:1 propensity score matching by PNI group.

| Variables | Low PNI (n = 223) | High PNI (n = 223) | SMD |
| --- | --- | --- | --- |
| Age | 68.5 (56.0-78.0) | 67.5 (55.5-77.5) | 0.042 |
| Male sex n(%) | 158 (70.9%) | 161 (72.2%) | 0.029 |
| Surgery, n(%) | 62 (27.8%) | 65 (29.1%) | 0.031 |
| Antibiotics, n(%) | 215 (96.4%) | 214 (96.0%) | 0.022 |
| Ventilator, n(%) | 88 (39.5%) | 85 (38.1%) | 0.028 |
| ICU Admission, n(%) | 102 (45.7%) | 99 (44.4%) | 0.027 |
| CRKP resistant phenotype, n(%) | 103 (46.2%) | 76 (34.1%) | 0.25 |

Note: Data are presented as median (interquartile range, IQR) or n (%). PNI, prognostic nutritional index; ICU, intensive care unit; CRKP, carbapenem-resistant *K. pneumoniae*; SMD, standardized mean difference. The low-PNI group was defined as PNI <42.1 and the high-PNI group as PNI ≥42.1. An SMD <0.1 indicates acceptable balance for matched baseline covariates. The CRKP phenotype was the outcome and was not included in the propensity score model; its SMD therefore reflects an outcome difference rather than residual imbalance of matched covariates.

Supplementary Table S5 summarizes the association between prespecified PNI strata and the CRKP phenotype in the total cohort. The high-PNI group (≥45) was the reference category.

Supplementary Table S5. Association between PNI strata and the CRKP phenotype in the total cohort (N=1,360).

| PNI strata | Total patients, n | Resistant phenotype, n  (%) | Unadjusted OR  (95% CI) | *P* value | Adjusted ORa  (95% CI) | *P* value |
| --- | --- | --- | --- | --- | --- | --- |
| High (≥45) | 515 | 135 (26.2%) | Reference | — | Reference | — |
| Medium (38–<45) | 416 | 182 (43.8%) | 2.19 (1.66–2.88) | <0.001 | 1.83 (1.37–2.45) | <0.001 |
| Low (≤38) | 429 | 217 (50.6%) | 2.88 (2.19–3.78) | <0.001 | 2.14 (1.60–2.87) | <0.001 |

Note: a Adjusted for age, recent antibiotic exposure, ICU admission, mechanical ventilation, and surgical admission. Strata were defined as high (≥45), medium (38–<45), and low (≤38), without overlap.

Supplementary Table S6 reports discrimination of a PNI-only model as a benchmark for the incremental value analysis.

Supplementary Table S6. Performance of the PNI-only model.

| **Analysis Scenarios** | **Derivation Cohort (n=987)** | **Temporal Internal Validation Cohort (n=373)** | *P* Value |
| --- | --- | --- | --- |
| Model 3: PNI Only | 0.632 | 0.621 | - |

Note: The PNI-only model is not intended for standalone clinical use; it is reported as a baseline comparator.

Supplementary Table S7 summarizes the admission-to-culture interval and specimen-source distribution according to the CRKP phenotype in the total cohort.

Supplementary Table S7. Admission-to-culture interval and specimen-source distribution according to the CRKP phenotype in the total cohort (N=1,360).

| **Variables** | **Total Cohort (n=1,360)** | **CSKP (n=826)** | **CRKP (n=534)** | *P* Value |
| --- | --- | --- | --- | --- |
| Admission-to-culture interval, days | 9.43 (4.46–20.52) | 7.22 (3.55–18.16) | 12.46 (6.29–23.38) | <0.001 |
| Specimen source |  |  |  | <0.001 |
| Sputum | 935 (68.8%) | 538 (65.1%) | 397 (74.3%) |  |
| Blood | 71 (5.2%) | 49 (5.9%) | 22 (4.1%) |  |
| Urine | 139 (10.2%) | 88 (10.7%) | 51 (9.6%) |  |
| Bronchoalveolar lavage fluid | 53 (3.9%) | 29 (3.5%) | 24 (4.5%) |  |
| Other | 162 (11.9%) | 122 (14.8%) | 40 (7.5%) |  |

Note: Admission-to-culture interval is presented as median (interquartile range) in days. The *P* value for specimen source is the global chi-square test across the five categories.

Supplementary Table S8. Extended multivariable logistic regression additionally adjusted for admission-to-culture interval and specimen source.

| **Variable** | **OR** | **95% CI** | *P* Value |
| --- | --- | --- | --- |
| Age, per year | 1.011 | 1.002–1.020 | 0.018 |
| PNI, per point | 0.967 | 0.954–0.981 | <0.001 |
| Recent antibiotic exposure | 6.094 | 2.447–15.176 | <0.001 |
| ICU admission | 2.075 | 1.561–2.759 | <0.001 |
| Mechanical ventilation | 1.231 | 0.873–1.736 | 0.236 |
| Surgical admission | 0.471 | 0.324–0.683 | <0.001 |
| Admission-to-culture interval, per 7 days | 1.035 | 0.985–1.087 | 0.177 |
| Blood vs sputum | 0.687 | 0.372–1.267 | 0.229 |
| Urine vs sputum | 0.754 | 0.472–1.205 | 0.238 |
| Bronchoalveolar lavage fluid vs sputum | 1.477 | 0.681–3.203 | 0.323 |
| Other vs sputum | 0.432 | 0.264–0.706 | 0.001 |

Note: Admission-to-culture interval was modeled per 7-day increase. Sputum was the reference specimen category. This extended model was fitted as a sensitivity analysis and does not replace the six-variable primary model reported in Table S1. The AUC was 0.718 in the derivation cohort and 0.703 in the temporal internal validation cohort.
